# Nomogram for Predicting Distant Metastasis of Pancreatic Ductal Adenocarcinoma: A SEER-Based Population Study

**DOI:** 10.3390/curroncol29110643

**Published:** 2022-10-28

**Authors:** Weibo Li, Wei Wang, Lichao Yao, Zhigang Tang, Lulu Zhai

**Affiliations:** 1 Department of General Surgery, Renmin Hospital of Wuhan University, Wuhan 430060, China; 2 Department of General Surgery, The First Affiliated Hospital of the University of Science and Technology of China, Hefei 230001, China

**Keywords:** pancreatic ductal adenocarcinoma, pancreatic cancer, distant metastasis, risk, prognosis

## Abstract

(1) Background: The aim of this study was to identify risk factors for distant metastasis of pancreatic ductal adenocarcinoma (PDAC) and develop a valid predictive model to guide clinical practice; (2) Methods: We screened 14328 PDAC patients from the Surveillance, Epidemiology, and End Results (SEER) database between 2010 and 2015. Lasso regression analysis combined with logistic regression analysis were used to determine the independent risk factors for PDAC with distant metastasis. A nomogram predicting the risk of distant metastasis in PDAC was constructed. A receiver operating characteristic (ROC) curve and consistency-index (C-index) were used to determine the accuracy and discriminate ability of the nomogram. A calibration curve was used to assess the agreement between the predicted probability of the model and the actual probability. Additionally, decision curve analysis (DCA) and clinical influence curve were employed to assess the clinical utility of the nomogram; (3) Results: Multivariate logistic regression analysis revealed that risk factors for distant metastasis of PDAC included age, primary site, histological grade, and lymph node status. A nomogram was successfully constructed, with an area under the curve (AUC) of 0.871 for ROC and a C-index of 0.871 (95% CI: 0.860–0.882). The calibration curve showed that the predicted probability of the model was in high agreement with the actual predicted probability. The DCA and clinical influence curve showed that the model had great potential clinical utility; (4) Conclusions: The risk model established in this study has a good predictive performance and a promising potential application, which can provide personalized clinical decisions for future clinical work.

## 1. Introduction

Pancreatic ductal adenocarcinoma (PDAC) is a highly aggressive malignant tumor characterized by metastatic susceptibility and high mortality, with a rising incidence in both economically developed and developing countries [1]. Worldwide, PDAC is the 12th most commonly diagnosed cancer and the seventh leading cause of cancer death in 2020 [2]. In the United States, PDAC is estimated to be the fourth leading cause of cancer-related deaths in 2022, and its mortality rate is maintaining an upward trend, with the disease projected to rank second in mortality among all cancer types by 2030 [3,4]. The five-year relative overall survival (OS) rate for the disease is about 11% and most patients die from peritoneal spread or distant metastases [3]. For metastatic PDAC, the five-year OS rate is only 2% [5]. The most common site of metastasis in PDAC is the liver, followed by the lung, bone, and brain [6,7,8]. Although several previous studies have evaluated the prognostic role and predictors of single-organ metastasis of PDAC, studies on the prediction of risk for distant metastasis of PDAC are still not available [6,9]. Metastasis of PDAC is affected by a variety of clinicopathological factors such as gender, age, race, primary site, grade, tumor size, and lymph node status [10]. Population-level estimates for the risk of distant metastases in PDAC patients are lacking, and the relationship between clinical-related factors and distant metastasis have not been well-elucidated. Therefore, in this study, we used data from patients diagnosed with PDAC in the Surveillance, Epidemiology, and End Results (SEER) cancer registry between 2010 and 2015 to analyze the impact of distant metastasis on the prognosis of PDAC patients, to identify risk factors for distant metastasis in PDAC, and to develop an effective predictive model that provides a guiding strategy for clinical practice.

## 2. Materials and Methods

### 2.1. Patients Selection

**Figure 1:**
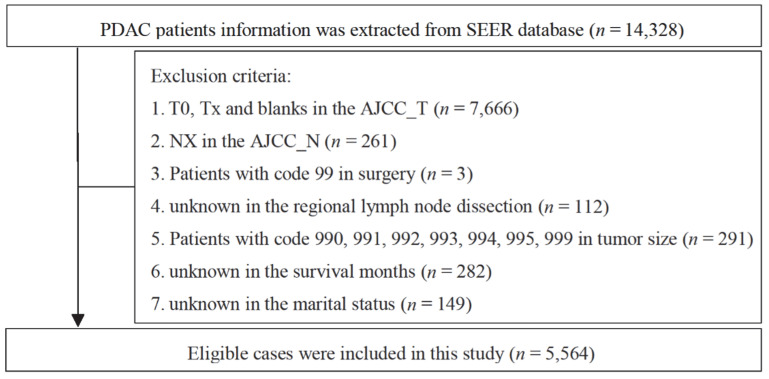
The flow chart of eligible patients’ selection in this study.

A retrospective cohort study was performed by extracting data from the SEER database, which is the authoritative cancer database in the United States, collecting cancer data from 18 population-based cancer registries in 14 states. “Incidence-SEER Research Plus Date, 13 Registries, November 2020 sub (2000–2018)” was employed as the data source. We selected cases through “Site code ICD-O-3/WHO 2008” and regarded “Pancreas” as the site of morbidity to determine the patient cohort of this study and extract patient-related information. Based on previous studies using the same database, we identified PDAC by the codes defined by “ICD-O-3 Hist/Behav, Malignant” including 8140 and 8500 [11] (8140: Adenocarcinoma, 8500: infiltrating duct carcinoma). Demographic and clinical data were extracted for every patient, including age, gender, race, primary site (defined encoding: C25.0: Head of pancreas, C25.1: Body of pancreas, C25.2: Tail of pancreas, C25.3: Pancreatic duct, C25.4: Islets of Langerhans, C25.7: Other specified parts of pancreas, C25.8: Overlapping lesion of pancreas, C25.9: Pancreas), tumor size, histologic grade, TNM stage (AJCC 7th edition), surgery, regional lymph node dissection, marital status, radiotherapy, chemotherapy and the survival data of the patients. Inclusion criteria were as follows: (1) age ≥ 18 years; (2) histologically confirmed disease; (3) definite distant metastasis status; (4) diagnosed in 2010–2015. The exclusion criteria were shown in Figure 1. Ultimately, 5564 PDAC patients were eligible for subsequent analysis. SEER*Stat software (version 8.3.9.2) was used to extract data. This study complies with the requirements of the Declaration of Helsinki. Since SEER data are publicly available, this study did not require approval from the institutional ethics committee and informed consent was waived.

### 2.2. Statistical Analysis

The software used for statistical analysis in this study included IBM SPSS (version 22.0), GraphPad Prism (version 8.0.1), and RStudio (version 1.4.1717.0). The applied R function packages included rms, readr, corrplot, glmnet, pROC, rmda, and ResourceSelection. The chi-square test or Fisher exact test were deployed to compare categorical variables between different groups. Lasso regression method was employed to reduce the risk of over-fitting of the model. The minimum partial likelihood deviance representing the complexity of the model was applied by the 10-fold cross-validation method. As a result, none of the independent variables were eliminated in the optimal case. Univariate logistic regression analyses were then performed, and variables with *p* values less than 0.05 were selected to enter a multivariate logistic regression model to determine the independent predictors for distant metastasis of PDAC. The nomogram was drawn based on the independent predictors. The Hosmer–Lemeshow (HL) test was used to assess the goodness-of-fit of the model. The accuracy and discrimination of the model was assessed by the area under the curve (AUC) of the ROC curve and the calculation of the consistency-index (C-index). The agreement between the predicted probability of the model and the actual probability was evaluated by a calibration curve. Furthermore, a decision curve analysis (DCA) and clinical influence curve were used to assess the clinical utility of the model. Finally, we analyzed the survival differences between patients with distant metastasis and without distant metastasis. In the survival analysis, the primary endpoints were OS and cancer-specific survival (CSS). Survival curves were plotted using Kaplan–Meier method and differences were compared using the log-rank test. *p* values for all analyses were bilateral, and *p* < 0.05 was considered statistically significant in this study.

## 3. Results

### 3.1. Characteristics of PDAC Patients

A total of 5564 patients with PDAC that met our criteria were included in the study. Among them, 1242 patients (22.3%) with distant metastasis. The rate of distant metastasis in PDAC patients decreased gradually with the increasing of age. The rate of distant metastasis was significantly lower in patients over 80 years of age than in those under 60 years of age. The incidence of distant metastasis was higher in men than in women. The incidence of distant metastases was significantly higher in blacks than in other ethnic groups. The incidence of distant metastasis of PDAC originating from the body and tail of the pancreas was more than twice that of PDAC originating from the head of the pancreas. The high incidence of distant metastasis of PADC was significantly associated with worse tumor differentiation and larger tumor size. More invasive surgery and radiotherapy significantly reduced the incidence of distant metastases of PDAC. Detailed demographic and clinicopathological characteristics of PDAC patients were summarized in Table 1.

### 3.2. Risk Factors for Distant Metastasis of PDAC

**Figure 2:**
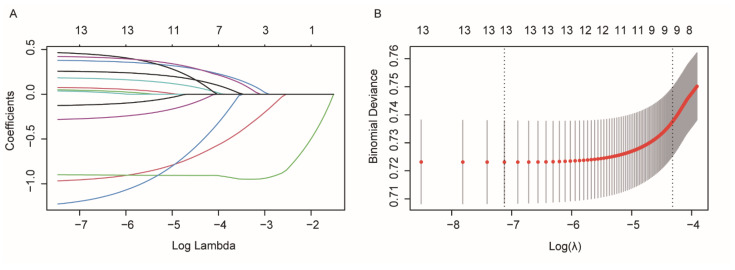
(**A**) The lasso regression is used to find the optimal lambda value. (**B**) Results of 10-fold cross-validation.

We used the lasso regression method to reduce the risk of over-fitting of the developed model, which compresses the partial factorial regression coefficients to zero. We performed 100 calculations using the glmnet package in RStudio software, and the results converged to the optimal solution at the 65th time (lambda = 0.00571). The degree of freedom corresponding to the optimal solution was 13. This means that none of the 13 independent variables included in this study were eliminated (Figure 2A). The mean-square error of the model at different lambda values was then checked through 10-fold cross-verification (Figure 2B). The lambda value corresponding to a standard error space with the minimum mean square error was taken as the optimal solution, and the regression coefficients of all independent variables at this time were checked. Subsequently, we included 13 independent variables in the univariate logistic regression analysis, and the variables with *p* ˂ 0.05 in the regression results were included in the multivariate logistic regression analysis. Finally, multivariate analysis showed that age ˂ 60 (OR = 2.481, 95% CI 1.906–3.238, *p* ˂ 0.001), age in 60–69 (OR = 2.076, 95% CI 1.626–2.657, *p* ˂ 0.001), age in 70–79 (OR = 1.790, 95% CI 1.397–2.299, *p* ˂ 0.001), the tumor located in the pancreatic body/tail (OR = 2.520, 95% CI 2.091–3.038, *p* ˂ 0.001), the tumor in other parts of the pancreas (OR = 1.622, 95% CI 1.293–2.033, *p* ˂ 0.001), moderately differentiated in the histological grade (OR = 1.652, 95% CI 1.247–2.201, *p* ˂ 0.001), poorly and undifferentiated in the histological grade (OR = 1.732, 95% CI 1.309–2.304, *p* ˂ 0.001), and N1 in the AJCC_N (OR = 1.708, 95% CI 1.431–2.041, *p* ˂ 0.001) were independent risk factor for distant metastases of PDAC. Partial pancreatectomy in the surgery (OR = 0.083, 95% CI 0.045–0.149, *p* ˂ 0.001), total pancreatectomy in the surgery (OR = 0.069, 95% CI 0.032–0.140, *p* ˂ 0.001), extended pancreatectomy in the surgery (OR = 0.076, 95% CI 0.026–0.195, *p* ˂ 0.001), and radiotherapy (OR = 0.303, 95% CI 0.195–0.456, *p* ˂ 0.001) were independent protective factor for distant metastases (Table 2).

### 3.3. Construction and Validation of the Nomogram

**Figure 3:**
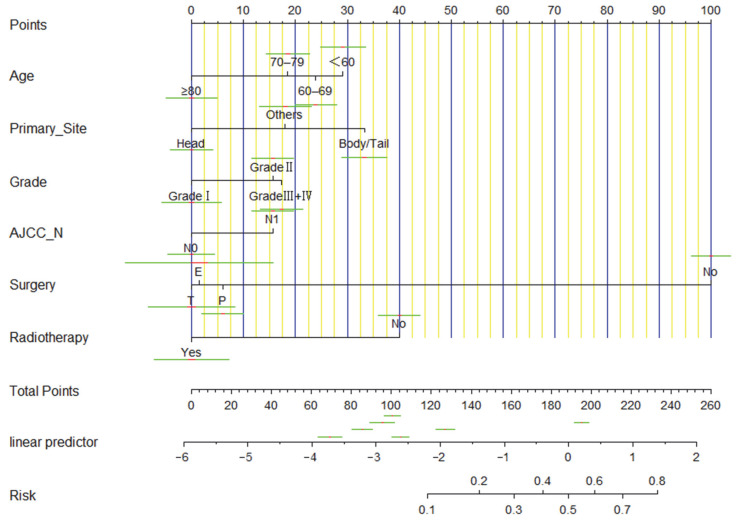
Nomogram to predict distant metastasis of PDAC. Others: including pancreatic duct, islets of Langerhans, other specified parts of pancreas, overlapping lesion of pancreas. T: total pancreatectomy; E: extended pancreatectomy; P: partial pancreatectomy.

**Figure 4:**
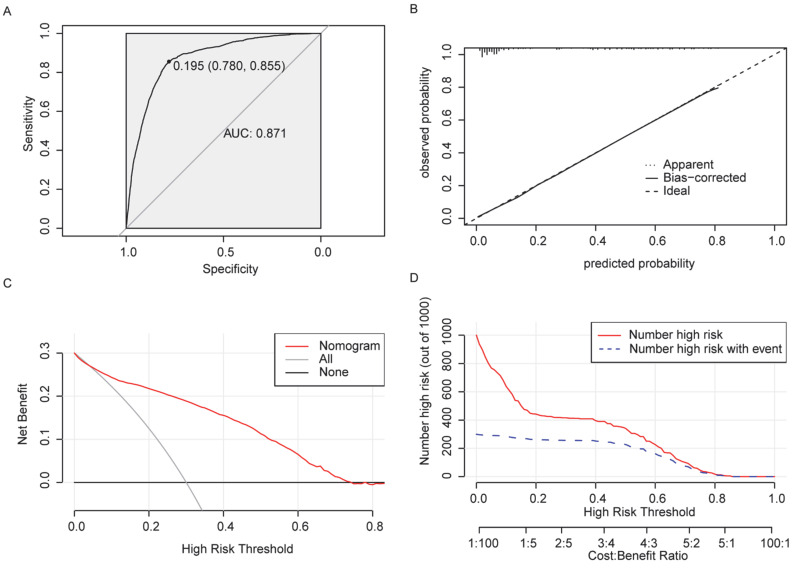
(**A**) Receiver operating characteristic curve. (**B**) Calibration curve. (**C**) Decision curve analysis. (**D**) Clinical influence curve.

Nomogram was established according to the significant variables determined by multivariate logistic regression analysis (Figure 3). The HL test showed a *p*-value of 0.394 (greater than 0.05), indicating a good fit of the model. The ROC curve of the nomogram was constructed, and the results revealed that the AUC value of the model was 0.871, its cut-off value was 0.195, and the corresponding specificity and sensitivity were 0.780 and 0.855, indicating that this model had good predictive performance (Figure 4A). The C-index of the nomogram was 0.871 (95% CI 0.860–0.882), suggesting a good discriminatory ability of the model. We further employed the calibration curve to evaluate the agreement between the probability of occurrence of events predicted by the model and the actual probability of occurrence of events using the bootstrap method 1000 times (Figure 4B). Finally, we plotted DCA and clinical influence curves to observe the clinical utility of the model. DCA indicated that the nomogram had a wide threshold probability range and a positive net return (Figure 4C). In the clinical impact curve (Figure 4D), the red curve represents the number of people classified as positive by the model at each threshold probability and the blue curve represents the number of true positive people at each threshold probability, showing that with the increase of threshold probability, the accuracy of the prediction model for distant metastasis of PDAC was closer to the true value.

### 3.4. Survival Analysis

**Figure 5:**
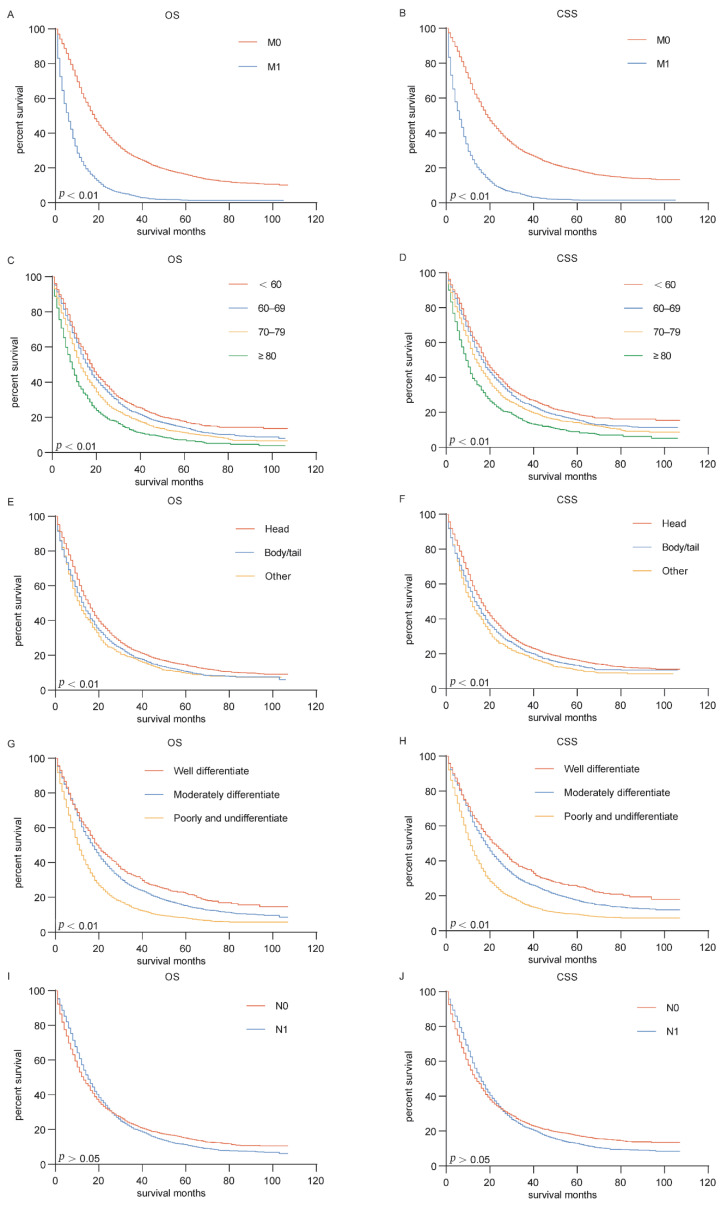
Kaplan-Meier survival curves for PDAC patients. (**A**,**B**) Effects of distant metastasis on OS and CSS. (**C**,**D**) Effects of age on OS and CSS. (**E**,**F**) Effects of primary site on OS and CSS. (**G**,**H**) Effects of grade on OS and CSS. (**I**,**J**) Effects of lymph node status on OS and CSS. OS, Overall survival; CSS, cancer specific survival.

Survival analysis was performed according to the situation of distant metastasis and different independent risk factors. The Kaplan–Meier curves of CSS and OS were shown in Figure 5. Median OS was 18 and 6 months and median CSS was 19 and 6 months in PDAC patients without and with distant metastases, respectively, with statistically significant differences between groups (Figure 5A,B; *p* < 0.01). Comparison of different age subgroups showed that advanced age reduced the survival of patients with PDAC. The median survival for both OS and CSS was 9 months in patients with PDAC aged 80 years or older, and in the age group below 60 years, the median survival for both OS and CSS was 18 months (Figure 5C,D). There was no significant difference in the survival of patients with PDAC originating from different sites of the pancreas (Figure 5E,F). Figure 5G,H showed that the survival of patients with well differentiated PDAC was significantly better than those with poorly differentiated and undifferentiated PDAC, with median OS of 20 and 11 months and median CSS of 22 and 11 months, respectively (*p* < 0.01). In addition, there was no statistically significant difference in survival between PADC patients with and without lymph node metastasis (Figure 5I,J).

## 4. Discussion

PDAC accounts for approximately 90% of all pancreatic tumors and is an extremely aggressive malignancy [12]. Although patients with PDAC receive anticancer treatment, the outcome is not satisfactory, and most PDAC patients die from peritoneal spread or distant metastasis [13]. The prediction of PDAC patients with a high probability of distant metastasis allows the development of more aggressive and precise treatment measures, which is of great relevance to improve survival. Therefore, the present study investigated risk factors and developed a valid prediction model for distant metastasis of PDAC. The findings showed that risk factors for distant metastasis of PDAC included young age, worse histological differentiation, body and tail tumors originating from the pancreas, and lymph node metastasis. The risk model established had good predictive performance and great potential application. The AUC values of the ROC curve and C-index showed that the model had good predictive performance and discriminatory ability. The calibration curve showed that the predicted probability of the model was in high agreement with the actual predicted probability. The DCA and clinical influence curve revealed that the model had great potential clinical utility.

The relationship between age and the aggressive progression of cancer has been demonstrated. Previous studies on thyroid cancer, breast cancer, and colorectal cancer revealed an inverse association between age and the risk of distant metastasis of malignant tumors [14,15,16,17]. In this study, we found that the risk of PDAC metastasis decreased with increasing age. The possible reason for the association of younger age with distant metastasis is the progressive degeneration of B- and T-lymphocytes with advancing age [18]. The adaptive immune system also undergoes significant disruption through more complex pathways, and age-related deterioration of the immune system may actually be protective by depriving the metastatic process of key immune cellular components [19]. In addition, aging inhibits cancer cell metastasis by altering the extracellular matrix through non-enzymatic glycosylation and reducing the activity of matrix-modifying proteases [18]. Survival analyses showed that younger patients with PDAC had longer survival time than older patients. The potential causes perhaps were younger patients usually have a more positive attitude toward therapies and have better physical condition to tolerant various treatment modalities. However, this study did not consider whether the elder patients received less systemic therapy than the younger patients. In addition, since elderly PDAC patients are in poorer physical condition, the probability of non-neoplastic death is higher, resulting in shorter survival and thus not enough time for distant metastases to occur, which is also a possible factor contributing to the low risk of distant metastases in elderly patients. Therefore, clinicians should pay special attention to whether patients under 60 years of age are at high risk of distant metastases, which may affect their prognosis. Yamaguchi et al. [20] showed that precancerous pancreatic cells could undergo latent metastasis at an early stage and remain latent in the host organ. Although these cells did not appear malignant at an early stage, what is surprising is that such distant metastases appeared before the development of the primary tumor site. Such early disseminated cells can develop in parallel with the primary tumor [20,21]. Therefore, the combined effect of age and latent metastasis of precancerous cells should be considered in young PDAC patients at an early stage, and aggressive management strategies should be given to minimize the chances of metastasis after treatment.

Due to the anatomical location, patients with PDAC in the body and tail of the pancreas usually have symptoms caused by biliary obstruction later than patients with PDAC in the head of the pancreas. Consequently, in clinical practice, PDAC in the body and tail of the pancreas is often detected with more advanced symptoms than PDAC in the head of the pancreas [22]. Previous studies have also demonstrated differences in biological behavior and molecular levels between PDAC in different locations of the pancreas [23]. PDAC originating from the body and tail of the pancreas is more aggressive since it is enriched for gene programs involved in tumor cell invasion and epithelial-to-mesenchymal transition and is characterized by a poor antitumor immune response [24]. A single-center retrospective study showed that the pattern of distant metastasis was significantly different in PDAC from the body and tail than in PDAC from the head of the pancreas [25]. PDAC located in the body and tail of the pancreas was larger in diameter and more prone to distant metastases [25]. A multicenter study similarly demonstrated that PDAC from the body and tail of the pancreas was significantly more aggressive than PDAC from the head of the pancreas [26]. The risk model in this study suggested that PDAC in the body and tail of the pancreas had a higher risk score for distant metastasis than PADC in the head of the pancreas. Our data demonstrated that there was no significant difference in survival time for patients with PDAC from different parts of the pancreas. It is important to explain that the survival analysis in this study was performed based on all patients with PADC, including those with and without distant metastasis. For patients with advanced PDAC, PDAC in the body and tail of the pancreas was more malignant than PDAC in the head of the pancreas [27]. In PDAC patients with stage I, survival rates were higher for cancers of the body and tail of the pancreas than for cancers of the head of the pancreas, but the opposite was true in patients with stage II to IV PDAC [22]. As a result, patients with middle to advanced PDAC in the body and tail of the pancreas have a lower survival rate and are more prone to metastasis. More attention should be paid to these patients.

Tumor grade correlates with the malignant biological behavior of cancer. Several studies have demonstrated that the degree of tumor differentiation was a determinant of distant metastasis [28,29]. In the present study, the proportion of poorly differentiated and undifferentiated tumors (26.8%) was significantly higher in PDAC patients with distant metastasis than in well differentiated (17.3%) and moderately differentiated (19.7%) tumors. Multivariate logistic regression analysis showed that the risk of distant metastasis of PDAC increased as the degree of differentiation decreased. Survival analysis showed that better differentiated tumors had significantly longer survival times.

The lymphatic tract is an important route for tumor cells to achieve metastasis. In colorectal cancer, more than one-third of distant metastasis may arise from lymph node metastasis, which share a common origin with distant metastasis [30,31]. Some tumor cells infiltrate into the lymphatic vessels and then colonize distant organs or tissues through the lymphatic circulation and continue to grow into metastases [32]. Han et al. [33] detected stem-like lymphatic circulating tumor cells from the thoracic duct that empties lymph directly into the circulation, suggesting a key role for the lymphatic system in mediating distant metastasis of cancer. The relationship between lymph node and distant metastasis has also been demonstrated in mouse cancer models by injecting tumor cells directly into lymphatic vessels and using photoswitchable tumor cell models [34,35]. Our data also showed that lymph node metastasis leads to an increased risk of distant metastasis of PDAC. Inconsistently, our data show that patients with lymph node metastases have a significantly lower proportion of distant metastases than patients without lymph node metastases. Accordingly, further large cohort studies are needed to reveal the relationship between lymph node metastasis and distant metastasis. Although regional lymph node dissection was not statistically significant in multivariate logistic regression, its demographics showed a significant reduction in the proportion of patients with distant metastasis.

We have to acknowledge the deficiencies of this research. First, the risk model for predicting distant metastasis of PDAC was based on patient information screened from a public database. Therefore, the effectiveness of external application of the model has yet to be tested. Second, the SEER data do not provide the specific dosing regimen of chemotherapy received by PDAC patients and whether they received neoadjuvant chemotherapy. This means that we might not demonstrated how neoadjuvant chemotherapy could change these results. Third, this database only included synchronous distant metastasis information, which meant that the patients developed metachronous metastasis lesions were not included in our study. Furthermore, the database only provided limited metastatic sites such as the bone, liver, lung and brain, and thus other sites of metastasis are not known. Finally, the nomogram was established based on a retrospective study and needs to be further validated in multicenter prospective cohorts and clinical trials. Despite these limitations in this retrospective study, the nomogram model has practical utility in different populations. Nomogram has been proved to be an efficient and instructive model that can effectively assist clinicians in providing personalized management.

## 5. Conclusions

In conclusion, our findings suggested that young age, poorer histological differentiation, body and tail tumors originating from the pancreas, and lymph node metastasis were risk factors for distant metastasis of PDAC. Furthermore, the present study successfully constructed a risk model with promising predictive value for distant metastasis of PDAC, which provides guidance for clinical practice in predicting distant metastasis of PDAC.

## Figures and Tables

**Table 1:** Demographic and clinicopathological characteristics of PDAC patients.

Variables	Total	No Metastasis	Metastasis	*p* Value
	5564	4322 (77.7%)	1242 (22.3%)	
Age (years)				0.008
˂60	1234 (22.2%)	921 (74.6%)	313 (25.4%)	
60–69	1851 (33.3%)	1430 (77.3%)	421 (22.7%)	
70–79	1659 (29.8%)	1315 (79.3%)	344 (20.7%)	
≥80	820 (14.7%)	656 (80.0%)	164 (20.0%)	
Sex				0.031
Female	2793 (50.2%)	2203 (78.9%)	590 (21.1%)	
Male	2771 (49.8%)	2119 (76.5%)	652 (23.5%)	
Race				˂0.001
Other ^a^	664 (11.9%)	536 (80.7%)	128 (19.3%)	
Black	589 (10.6%)	421 (71.5%)	168 (28.5%)	
White	4311 (77.5%)	3365 (78.1%)	946 (21.9%)	
Primary site				˂0.001
Head	3590 (64.5%)	3057 (85.2%)	533 (14.8%)	
Body/tail	1296 (23.3%)	823 (63.5%)	473 (36.5%)	
Other ^b^	678 (12.2%)	442 (65.2%)	236 (34.8%)	
Grade				˂0.001
Well differentiated	579 (10.4%)	479 (82.7%)	100 (17.3%)	
Moderately differentiated	2733 (49.1%)	2195 (80.3%)	538 (19.7%)	
Poorly and undifferentiated	2252 (40.5%)	1648 (73.2%)	604 (26.8%)	
AJCC ^c^_T				˂0.001
T1	241 (4.3%)	203 (84.2%)	38 (15.8%)	
T2	851 (15.3%)	518 (60.9%)	333 (39.1%)	
T3	3628 (65.2%)	3114 (85.8%)	514 (14.2%)	
T4	844 (15.2%)	487 (57.7%)	357 (42.3%)	
AJCC_N				˂0.001
N0	2525 (45.4%)	1821 (72.1%)	704 (27.9%)	
N1	3039 (54.6%)	2501 (82.3%)	538 (17.7%)	
Surgery				˂0.001
No	2039 (36.6%)	974 (47.8%)	1065 (52.2%)	
Partial pancreatectomy	2946 (53.0%)	2794 (94.8%)	152 (5.2%)	
Total pancreatectomy	436 (7.8%)	417 (95.6%)	19 (4.4%)	
Extended pancreatectomy	143 (2.6%)	137 (95.8%)	6 (4.2%)	
Dissected lymph nodes ^d^ (*n*)				˂0.001
None	2064 (37.1%)	1010 (48.9%)	1054 (51.1%)	
1–3	162 (2.9%)	140 (86.4%)	22 (13.6%)	
≥4	3338 (60.0%)	3172 (95.0%)	166 (5.0%)	
Radiotherapy				˂0.001
No	4465 (80.2%)	3250 (72.8%)	1215 (27.2%)	
Yes	1099 (19.8%)	1072 (97.5%)	27 (2.5%)	
Chemotherapy				0.113
No	1620 (29.1%)	1236 (76.3%)	384 (23.7%)	
Yes	3944 (70.9%)	3086 (78.2%)	858 (21.8%)	
Tumor size (cm)				˂0.001
˂2	446 (8.0%)	396 (88.8%)	50 (11.2%)	
2–4	3292 (59.2%)	2734 (83.0%)	558 (17.0%)	
˃4	1826 (32.8%)	1192 (65.3%)	634 (34.7%)	
Marital status				0.318
Married	3472 (62.4%)	2712 (78.1%)	760 (21.9%)	
Unmarried	2092 (37.6%)	1610 (77.0%)	482 (23.0%)	

^a^: Including Asian, Pacific Islander, and American Indian/Alaska Native; ^b^: Including pancreatic duct, islets of Langerhans, other specified parts of pancreas, overlapping lesion of pancreas; ^c^: American Joint Committee on Cancer; ^d^: Regional lymph nodes have been removed by surgery; unmarried: Including divorced, separated, single, unmarried or domestic Partner, Widowed.

**Table 2:** Univariate and multivariate logistic regression analyses for risk factors associated with distant metastasis of PDAC.

Variables	Univariate Analysis		Multivariate Analysis	
	OR (95% CI)	*p* Value	OR (95% CI)	*p* Value
Age (years)				
≥80	Reference		Reference	
˂60	1.359 (1.099–1.686)	0.005	2.481 (1.906–3.238)	˂0.001
60–69	1.178 (0.963–1.445)	0.114	2.076 (1.626–2.657)	˂0.001
70–79	1.046 (0.851–1.291)	0.670	1.790 (1.397–2.299)	˂0.001
Sex				
Female	Reference		Reference	
Male	1.149 (1.013–1.304)	0.031	1.117 (0.952–1.310)	0.174
Race				
Other	Reference		Reference	
Black	1.671 (1.286–2.176)	˂0.001	1.214 (0.875–1.685)	0.246
White	1.177 (0.961–1.451)	0.120	1.172 (0.910–1.515)	0.223
Primary site				
Head	Reference		Reference	
Body/tail	3.296 (2.849–3.814)	˂0.001	2.520 (2.091–3.038)	˂0.001
Other	3.062 (2.549–3.674)	˂0.001	1.622 (1.293–2.033)	˂0.001
Grade				
Well differentiated	Reference		Reference	
Moderately differentiated	1.174 (0.932–1.492)	0.181	1.652 (1.247–2.201)	˂0.001
Poorly and undifferentiated	1.756 (1.394–2.230)	˂0.001	1.732 (1.309–2.304)	˂0.001
AJCC_T				
T1	Reference		Reference	
T2	3.434 (2.391–5.050)	˂0.001	1.239 (0.709–2.184)	0.454
T3	0.882 (0.623–1.280)	0.492	0.714 (0.418–1.229)	0.220
T4	3.916 (2.728–5.756)	˂0.001	0.626 (0.360–1.094)	0.098
AJCC_N				
N0	Reference		Reference	
N1	0.556 (0.490–0.632)	˂0.001	1.708 (1.431–2.041)	˂0.001
Surgery				
No	Reference		Reference	
Partial pancreatectomy	0.050 (0.041–0.060)	˂0.001	0.083 (0.045–0.149)	˂0.001
Total pancreatectomy	0.042 (0.025–0.065)	˂0.001	0.069 (0.032–0.140)	˂0.001
Extended pancreatectomy	0.040 (0.016–0.083)	˂0.001	0.076 (0.026–0.195)	˂0.001
Dissected lymph nodes (n)				
None	Reference		Reference	
1–3	0.151 (0.093–0.233)	˂0.001	0.817 (0.444–1.482)	0.510
≥4	0.050 (0.042–0.060)	˂0.001	0.647 (0.358–1.211)	0.161
Radiotherapy				
No	Reference		Reference	
Yes	0.067 (0.045–0.097)	˂0.001	0.303 (0.195–0.456)	˂0.001
Chemotherapy				
No	Reference			
Yes	0.895 (0.781–1.027)	0.113		
Tumor size (cm)				
˂2	Reference		Reference	
2–4	1.616 (1.199–2.223)	0.002	1.004 (0.646–1.594)	0.985
˃4	4.212 (3.120–5.803)	˂0.001	1.544 (0.983–2.476)	0.065
Marital status				
Married	Reference			
Unmarried	1.068 (0.938–1.216)	0.318		

## Data Availability

The original contributions presented in the study are included in the article. Further inquiries can be directed to the corresponding authors.
